# Need for uniqueness moderates the effectiveness of different types of scarcity appeals

**DOI:** 10.3389/fpsyg.2022.890350

**Published:** 2022-11-25

**Authors:** Yan Wang, Shuhong Kong, Meng Li, Lin Liu

**Affiliations:** Shanghai Key Laboratory of Mental Health and Psychological Crisis Intervention, School of Psychology and Cognitive Science, East China Normal University, Shanghai, China

**Keywords:** demand-based scarcity, scarcity appeals, supply-based scarcity, need for uniqueness, perceived uniqueness

## Abstract

Marketers frequently implement scarcity messages in promoting their products. Scarcity due to demand and scarcity due to supply have both been found to influence consumers’ product evaluations positively. However, the differential effects of these two types of scarcity messages have been understudied. Study 1 manipulated scarcity appeals type and need for uniqueness orthogonally and examined their effects on purchase intention. Study 2 manipulated scarcity appeals type and tested its effect on perceived uniqueness. Study 3 manipulated scarcity appeals type and tested the moderated mediation model that perceived uniqueness mediated the interactive effects of scarcity type and need for uniqueness on purchase intention. Across three studies, we find that consumers perceive supply-based scarcity products as more unique than demand-based scarcity products. Consequently, in comparison with demand-based scarcity messages, supply-based scarcity messages increase purchase intention for consumers with high need for uniqueness. In contrast, these messages decrease purchase intention for consumers with low need for uniqueness. Our findings contribute to the research on scarcity appeals, uniqueness perception, and need for uniqueness. Our research also suggests that marketers need to implement different types of scarcity appeals to convey uniqueness information and to attract different consumers.

## Introduction

Scarcity promotion, which is defined as a marketing strategy that emphasizes the limited availability (in quantity or time) of a specific product or event (Ku et al., 2012), is ubiquitous in everyday lives. People prefer scarce products over abundant ones, and scarcity preference emerges even as early as 6 years old (John et al., 2018). Marketers often artificially create scarcity of products and services and highlight their limited availability or even out-of-stock status (Cialdini, 2001). A liquor factory may limit the number of cases it produces each year, such that consumers need to wait months to buy one. An online shopping site may display the number of people who have booked a product, revealing that the product is low in stock. Studies have also shown that product scarcity may increase perceived value and purchase intentions for products (Gierl and Huettl, 2010; Jang et al., 2015).

Researchers have proposed that scarcity preference is multiply determined, such as the feelings of distinctiveness and uniqueness that a scarce item provides (Snyder, 1992). In addition, consumers use scarcity as a heuristic cue to infer high product quality and value (Lynn and Bogert, 1996). However, a distinction should be made between two types of product scarcity: demand-based scarcity and supply-based scarcity (Gierl and Huettl, 2010). Although both scarcity types lead to favorable product evaluations, the underlying mechanisms may differ. In addition, the effects of the distinct types of scarcity depend on situational and individual differences (Ku et al., 2012).

While research has revealed a great deal about positive effects of scarcity appeals, recent literature has suggested potential downsides of scarcity appeals (Biraglia et al., 2021). In addition, researchers have argued that scarcity appeals should be tailored to the specific consumer segment where they would be most effective (Ang et al., 2021). Yet relatively little scholarly research has examined the nuances between different types of scarcity appeals and how they influence various consumer segments. From a practical perspective, this topic is of interest to marketers who are contemplating how to segment and target potential consumers with customized scarcity promotions.

In the current research, we propose and provide evidence for when and how supply-based scarcity (vs. demand-based scarcity) leads to stronger purchase intention. Study 1 showed that scarcity due to supply leads to higher purchase intention than scarcity due to demand for people with high need for uniqueness. In contrast, scarcity due to supply leads to lower purchase intention than scarcity due to demand for people with low need for uniqueness. The instances of supply-based scarcity and demand-based scarcity were pretested and matched in scarcity level. Need for uniqueness was manipulated. Study 2 showed that supply-based scarcity leads to higher level of perceived product uniqueness than demand-based scarcity. Study 3 tested a whole moderated mediation model and showed that perceived uniqueness mediates the interactive effects of scarcity type and need for uniqueness on purchase intention. Need for uniqueness was measured as a personality trait.

## Theoretical background

### Demand-based versus supply-based scarcity

Scarcity refers to a real or perceived lack of certain resources that individuals use to meet their needs and desires (Hamilton et al., 2019). It may take a wide variety of forms, such as scarcity of money, time, food, and products (Cannon et al., 2019; Liang et al., 2021). Research has shown that scarcity attracts individuals’ attentional focus toward a certain resource that is lacking (Mullainathan and Shafir, 2013; de Bruijn and Antonides, 2022). In addition to shifting attentional focus, the experience of lacking sufficient recourses triggers a general sense of scarcity, which regulates people to accommodate the discrepancy between their current and desired states (Cannon et al., 2019).

Marketers may strategically create scarcity by limiting the quantity or the time availability of products (Cialdini, 2001). Such scarcity is often communicated to consumers through scarcity appeals, which entail the communication of the limited availability of advertised promotions (Shi et al., 2020). These strategies are often effective such that product scarcity increases perceived value and desire for the product (Gierl and Huettl, 2010). However, an important distinction should be made between two types of product scarcity: scarcity due to high demand (e.g., “nearly sold out”) and scarcity due to low supply (e.g., “limited editions”). Consumers tend to make different inferences about product scarcity due to excess demand versus restricted supply; consequently, consumers respond differently to demand versus supply scarcity appeal (Roy and Sharma, 2015).

Scarcity due to excessive demand leads consumers to infer that a product is popular because others have already bought the product (Roy and Sharma, 2015). Furthermore, inference about popularity may readily activate the scarcity-is-good heuristic, which can be described as “If everyone is trying it, it must be good” (Worchel et al., 1975). As a result, when product knowledge is limited, consumers adopt products that others acquire and are scarce for that reason. Thus, demand-based scarcity appeals may be used to signal popular consumption, especially for people who seek conformity (Roy and Sharma, 2015). Scarcity due to limited supply implies that the number of potential consumers of the product is restricted from the beginning because marketers control product distribution decisions (Aguirre-Rodriguez, 2013). Consequently, acquiring a scarce product of this type can express uniqueness and signal high social status (Gierl and Huettl, 2010). Thus, supply-based scarcity is especially suitable for luxury goods and may readily serve as status signals (Jang et al., 2015).

### Scarcity type and purchase intention: The moderating role of need for uniqueness

Previous work suggests that whether scarcity has positive effects on purchase intentions depends on consumption contexts and consumer characteristics. An evolutionary approach proposes and provides evidence that romantic desire drives scarcity appeals to be more persuasive and fear makes scarcity appeals less persuasive (Griskevicius et al., 2009). Developmental research found that scarcity preference only appears in an individualist society but not in a collectivist society (Diesendruck et al., 2019). Although these studies did not specify the concrete type of the scarcity, the source of scarcity is the low supply rather than the high demand. In the luxury market, supply-based scarcity (i.e., limited edition) advertising is effective only for socially visible (vs. invisible) luxury products and for consumers in emotional (vs. cognitive) consumption contexts (Tseng et al., 2021). In the food and beverage industry, limited edition packages lead to lower purchase intentions unless people have a high need for uniqueness (Dörnyei and Lunardo, 2021). However, the abovementioned studies did not compare the two types of scarcity.

A comparison of the effects of demand- versus supply-based scarcity demonstrates that consumers’ responses depend on product types, consumption target, and consumer characteristics. Supply scarcity appeals are more effective in creating positive consumer attitudes toward conspicuous products, while demand scarcity appeals are more effective for non-conspicuous products (Gierl and Huettl, 2010). In the online retailing context, when purchasing for oneself, supply-driven scarcity cues outperform popularity cues (which imply high demand) in eliciting purchase intentions; conversely, when purchasing for someone else, popularity cues are more effective (Wu and Lee, 2016). Prevention-focused consumers are more likely to purchase a scarce product due to demand than due to supply, whereas promotion-focused consumers are more likely to purchase a scarce product due to supply than due to demand (Ku et al., 2012). In an online booking context, demand scarcity appeal leads to higher purchase intention than supply scarcity appeal among consumers with a high sense of power; in contrast, such a difference is attenuated among consumers with a low sense of power (Huang et al., 2020). Most relevant to the current research, consumers with low levels of need for uniqueness prefer demand over supply scarcity appeal, whereas consumers with high levels of need for uniqueness prefer supply over demand scarcity appeal (Roy and Sharma, 2015).

Need for uniqueness is defined as the desire to pursue differentiation from others, thereby motivates consumers to prefer products that promote distinctiveness (Snyder, 1992). Limited supply conveys the information that few people can acquire the product, irrespective of the number of consumers who are craving for it. Thus, scarce products due to limited supply can convey one’s distinctiveness, which is especially appealing to consumers’ need for uniqueness (Gierl and Huettl, 2010). As mentioned above, Roy and Sharma (2015) found that need for uniqueness moderates the relative effectiveness of demand versus supply scarcity. Thus, we aim to conceptually replicate the findings of Roy and Sharma (2015). Specifically, we hypothesize that:

Need for uniqueness moderates the effect of such that supply scarcity is more effective than demand scarcity for people with high need for uniqueness whereas demand scarcity is more effective than supply scarcity for people with low need for uniqueness (H1).

We extended the previous research in three ways. First, we matched both the objective and subjective scarcity level between the two types of scarcity appeals. Second, we manipulated need for uniqueness in addition to measuring it as a personality trait. Third, we further examined the mechanism of perceived product uniqueness.

### The mediating role of perceived uniqueness

Scarcity preference seems inconsistent with the premise of economic rationality because consumers act on the perception of irrelevant context cues (scarcity) and not of fundamental cues (objective attributes of the product, such as quality; Mittone and Savadori, 2009). In addition, a robust preference for scarce resources can be observed as early as age 5 years old (Ferera et al., 2020). Researchers propose several mechanisms through which scarcity is expected to exert a positive effect on consumers. For instance, commodity theory suggests that people value a commodity more when it is difficult to obtain or is even unavailable (Lynn, 1991). Meanwhile, signaling theory suggests that limited availability can signal high quality (Stock and Balachander, 2005) and/or high status (Gierl and Huettl, 2010) to potential consumers. However, commodity theory and signaling theory may not fully explain scarcity preference because it emerges at an age when individuals are incapable of understanding how market dynamics affect object value (Ferera et al., 2020) and even when scarcity is detached from any status symbol effect (Mittone and Savadori, 2009).

Another account links scarcity preference to self-related motivations, in particular, people’s need for some degree of uniqueness (He et al., 2010). When acquiring a scarce product, consumers perceive themselves as distinct from the mass. However, the uniqueness explanation may apply to supply-based scarcity better than demand-based scarcity (Diesendruck et al., 2019). Demand scarcity occurs when, even though the supply is abundant, the demand is so great that few of the products are left. Thus, demand scarcity implies that the product is highly popular and is less likely to satisfy the need for uniqueness (Roy and Sharma, 2015). In contrast, supply scarcity implies that the number of potential consumers of a product is restricted from the beginning of the market process (Gierl and Huettl, 2010). Thus, possessing such exclusive products can achieve and express uniqueness. Empirical research suggests that scarcity cues lead to higher level of perceived product uniqueness compared to popularity cues (Wu and Lee, 2016). In the previous research, popularity cues implied high demand, though whether the product is scarce was not clearly pointed out. Based on these propositions and findings, we hypothesize that:

People perceive greater uniqueness for supply-based scarcity than for demand-based scarcity (H2).

We extended the previous work (Wu and Lee, 2016) by holding the scarcity level constant such that the observed differences between conditions could only be attributed to scarcity type rather than the extent of scarcity.

Although people have a need to differentiate themselves from others, they also need to maintain a certain level of similarities (He et al., 2010). In addition, some consumers value exclusivity or uniqueness (i.e., snobs), whereas others value conformity (i.e., followers; Amaldoss and Jain, 2005). Consequently, supply scarcity and the proposed higher uniqueness perception may not always increase purchase intention. High uniqueness perception may increase or decrease purchase intention, which depends on consumers’ need for uniqueness (Roy and Sharma, 2015). Thus, we propose the following hypothesis:

The effect of scarcity type on purchase intention will be mediated by perceived uniqueness and then moderated by need for uniqueness (H3).

Specifically, supply-based (vs. demand-based) scarcity messages increase uniqueness perception, while perceived uniqueness increases purchase intention for consumers with high need for uniqueness. However, this uniqueness perception reduces purchase intention for consumers with low need for uniqueness. Prior work provides indirect evidence for our hypothesis. However, no study has tested the whole model directly.

### Overview of studies

Three studies tested the hypotheses. We piloted the two types of scarcity appeals to ensure they led to the same level of scarcity perception. Study 1 manipulated the need for uniqueness *via* a sentence unscrambling task and examined the interactive effects of supply- versus demand-based scarcity messages and need for uniqueness on purchase intention (H1). Study 2 compared the effects of supply- and demand-based scarcity messages on perceived uniqueness (H2). Study 3 examined the effect of scarcity type on purchase intention *via* perceived uniqueness while using measured need for uniqueness as the moderator of the effect of perceived uniqueness on purchase intention (H3). We used the PROCESS macro for SPSS to allow for mediation testing with bootstrapping; Model 15 is used for moderation and second-stage moderated mediation (Hayes, 2013).

## Study 1

Study 1 manipulated scarcity appeals and need for uniqueness orthogonally and tested the moderating role of need for uniqueness in the effect of scarcity type on purchase intention.

### Method

#### Participants and design

*A priori* power showed that a sample of 128 participants could successfully detect a moderate interaction effect of *f* = 0.25 (Faul et al., 2007). Out of the 137 participants who were recruited around the university campus, 11 were excluded for failing a manipulation check (see below). A final sample of 126 participants (88 women, aged 18–35 years old) were included. The study used a scarcity type (demand-based vs. supply-based) × need for uniqueness (high vs. control) between-subjects design. In this study, the need for uniqueness was manipulated as a situational factor by a priming task. Then, the participants were randomly assigned to one of four conditions.

#### Procedure and measures

The participants completed a task intended to manipulate the need for uniqueness. Then, they read a scenario describing a scarce product (demand-based vs. supply-based) and answered the manipulation check questions. Next, participants rated their purchase intention. Lastly, participants completed a demographic questionnaire. Across studies, participants’ gender never qualified the effect of scarcity type, and thus are not discussed.

##### Manipulation of need for uniqueness

A sentence-unscrambling task (Srull and Wyer, 1980) was developed and used to prime a situational need for uniqueness. Each experimental condition had 11 sets of out-of-order phrases. Participants in the high-uniqueness condition were asked to form meaningful sentences about uniqueness in consumption from the scrambled phrases (e.g., “privileged products desirable are to me”). In the control condition, participants formed 11 sentences unrelated to uniqueness (e.g., “dolls her daughter has”). To examine the effectiveness of the priming task, a pilot test (*N* = 43) was conducted. Participants rated their need for uniqueness *via* three items on seven-point scales (from 1 = *not at all agree* to 7 = *extremely agree*; “I feel like obtaining scarce and special products,” “Elite appeal in products is important to me,” and “Right now, need for uniqueness in consumption is at the top of my mind;” 𝛼 = 0.71; adapted from Lynn and Harris, 1997) after they finished the priming task. Participants in the high-uniqueness condition (*M* = 15.00, *SD* = 5.41) reported a greater need for uniqueness than those in the control condition (*M* = 11.29, *SD* = 3.98), *t*(41) = 2.56, *p* = 0.01, *d* = 0.78, thus showing that the manipulation was successful.

##### Piloted scarcity appeals

Scarcity type was manipulated with hypothetical consumption scenarios adapted from Gierl and Huettl (2010). Participants imagined thinking about buying a pair of fancy shoes in a store. Participants further imagined that the store only had one pair of his/her size left (i.e., the shoes were scarce). In the demand-based scarcity condition, participants were told that “because of the popularity of the shoes, they sell very well, and only one pair of this size is available.” In the supply-based scarcity condition, participants were told that “because the shoes are limited edition, only one pair of each size is available.”

Afterward, participants completed a manipulation check for the scarcity type manipulation, indicating “What is the reason for the scarcity of these shoes?” and selected from “*excess demand*” or “*limited supply*” (Ku et al., 2012). Any participant who selected the wrong option was excluded from the analysis (*N* = 11). To test whether the perceived scarcity level was similar across conditions, participants also responded to “How do you rate the scarcity level of these shoes in the current scenario?” on a seven-point scale (1 = *very inadequate* to 7 = *very adequate*).

A pilot test (*N* = 64) showed that the scenario manipulation worked. No significant difference was observed between the perceived scarcity level reported by the supply-based (*M* = 1.61, *SD* = 1.03) and demand-based group (*M* = 1.52, *SD* = 0.77), *t*(62) = 0.39, *p* = 0.70, *d* = 0.09. In addition, 94% of participants in the supply-based group selected “*limited supply*” as the reason for scarcity; 90% of participants in the demand-based group selected “*excess demand*” as the reason. The results demonstrated that participants determined that the reason for scarcity was consistent with manipulation and perceived that the two scarcity conditions insufficient similarly. Thus, the scenario manipulations had the intended effect.

##### Purchase intention

Purchase intention was measured by three items on seven-point scales (Dodds et al., 1991; Aggarwal et al., 2011; “the possibility that you will buy these shoes,” “the possibility that you will consider purchasing these shoes” “your willingness to buy these shoes”; 1 = *not likely to purchase at all*, 7 = *very likely to purchase*; 𝛼 = 0.91).

### Results

#### Manipulation check of scarcity type

A 2 × 2 analysis of variance (ANOVA) with perceived scarcity as the dependent variable showed that the main effects of scarcity appeal (*F*(1, 122) = 0.78, *p* = 0.379, *η*_p_^2^ = 0.006) and need for uniqueness were not significant (*F*(1, 122) = 1.23, *p* = 0.269, *η*_p_^2^ = 0.01). The interaction between the two factors was also not significant (*F*(1, 122) = 1.23, *p* = 0.269, *η*_p_^2^ = 0.01). The results indicated that scarcity type manipulation did not affect participants’ perception of scarcity, which had a similar level across conditions. Furthermore, the participants in each condition correctly identified the scarcity reason, respectively. Hence, the manipulation of scarcity appeal types was successful.

#### Hypothesis testing

As predicted, ANOVA analysis (scarcity appeal × need for uniqueness) with purchase intention as the dependent variable indicated that the interaction between the two independent variables was marginally significant (*F*(1, 122) = 8.32, *p* = 0.05, *η*_p_^2^ = 0.064). Meanwhile, the effects of scarcity appeal and need for uniqueness on purchase intention were nonsignificant (*p*s > 0.05).

**Figure 1:**
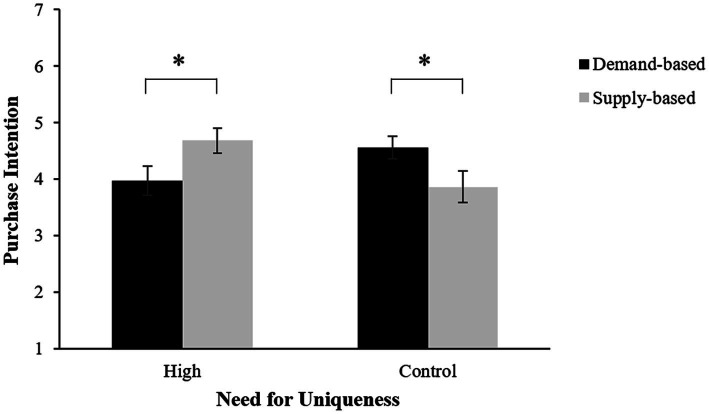
The effects of scarcity appeal and need for uniqueness on purchase intention. Error bars represent standard errors. *p < 0.05.

Contrast analysis found that participants in the uniqueness condition were more inclined to purchase a product in the supply-based scarcity condition (*M* = 4.68, *SD* = 1.26) than in the demand-based scarcity condition (*M* = 3.97, *SD* = 1.44; *t*(60) = −2.06, *p* = 0.043, *d* = 0.52). In the control group, the pattern was exactly reversed (*M*_demand-based_ = 4.56, *SD*_demand-based_ = 1.12; *M*_supply-based_ = 3.86, *SD*_supply-based_ = 1.60; *t*(62) = 2.02, *p* = 0.048, *d* = 0.51; see Figure 1).

### Discussion

The results from Study 1 confirmed the moderating role of need for uniqueness in the effect of the scarcity appeal on purchase intention. When consumers’ need for uniqueness was primed by sentence-unscrambling manipulation (vs. control), their purchase intentions for the supply-based scarcity product were higher than the demand-based scarcity product. These results supported H1.

## Study 2

Study 2 examined the effects of supply- versus demand-based scarcity appeal type on perceived uniqueness. This study served as a basis for the proposed mediating role of perceived uniqueness in the effect of scarcity type on purchase intention.

### Method

#### Participants and design

*A priori* power analyses showed that a sample of 102 participants could detect a moderate effect size of *d* = 0.50 (Faul et al., 2007). A total of 102 participants (36 women, aged 18–35 years old) were recruited from the university campus. They were assigned randomly to either the supply-based scarcity condition or the demand-based scarcity condition.

#### Procedure and measures

The participants viewed one of two scenarios describing a scarce product (supply-based vs. demand-based) and completed two items about manipulation check, as in Study 1. Afterward, the participants needed to report their perceived uniqueness in different types of scarcity appeal.

##### Perceived uniqueness

Perceived uniqueness was measured by a two-item scale adapted from Farwell and Wohlwend-Lloyd (1998) (“I perceived these shoes as highly unique”; “possessing these shoes make me distinct from other people”; 1 = *strongly disagree*, 7 = *strongly agree*; 𝛼 = 0.75).

### Results

#### Manipulation check of scarcity type

An independent sample *t*-test showed that participants perceived that the products were inadequate and the scarcity level of two conditions had no significant difference (*M*_supply-based_ = 1.57, *SD*_supply-based_ = 0.94; *M*_demand-based_ = 1.27, *SD*_demand-based_ = 0.60; *t*(100) = 1.88, *p* = 0.064). In addition, all of them selected the correct scarcity reason in line with the manipulation. The above results indicated that the manipulations had the intended effect.

#### Hypothesis testing

The results of an independent sample *t-*test on perceived uniqueness scores revealed a significant main effect of scarcity appeal (*t*(100) = 4.17, *p* < 0.001, *d* = 0.83). Specifically, participants perceived greater uniqueness for the supply-based scarce product (*M* = 5.27, *SD* = 1.28) than the demand-based scarce product (*M* = 3.98, *SD* = 1.81).

### Discussion

Study 2 tested the effect of scarcity appeal type on peoples’ perceived uniqueness. Consumers perceived the product as more unique when the scarce product was supposed to be supply based than demand based, thus supporting H2.

## Study 3

On the basis of the findings of Studies 1 and 2, Study 3 aimed to test the overall moderated mediation model, which expected that the need for uniqueness would moderate the indirect effect of scarcity type on purchase intention through perceived uniqueness.

### Method

#### Participants and design

*A priori* power showed that a sample of 199 participants could successfully detect a small-to-moderate interaction effect of *f* = 0.20 (Faul et al., 2007). A total of 200 participants (124 women, 26.5% aged 18–26 years old, 47% aged 27–35 years old, 26.5% aged over 35) were recruited from an online participant pool^1^ to take part in a single-factor (scarcity appeal: demand-based vs. supply-based) between-subjects experiment. The need for uniqueness was measured as an individual disposition continuous variable.

#### Procedure and measures

First, participants were informed to indicate their need for uniqueness. Then, they read a scarcity scenario centered on the same product and completed a manipulation check, as in Study 1. Next, the participants rated their perceived uniqueness (as in Study 2) and purchase intention (as in Study 1). Finally, we collected the demographics.

##### Need for uniqueness

The four items used to measure a participant’s need for uniqueness (“I like unique and scarce products”; “I enjoy products more when only a few people possess them”‘; “I enjoy having things that others do not”; “I often try to avoid products or bands that can be easily duplicated”; 1 = *strongly disagree*, 7 = *strongly agree*; 𝛼 = 0.77) were adapted from Lynn and Harris (1997).

### Results

#### Manipulation check of scarcity type

As expected, no significant difference was observed in the perceived scarcity between participants in the supply-based and demand-based conditions (*M*_supply-based_ = 1.21, *SD*_supply-based_ = 0.45; *M*_demand-based_ = 1.13, *SD*_demand--based_ = 0.34; *t*(198) = 1.35, *p* = 0.18, *d* = 0.20). Meanwhile, all participants selected the scarcity reason that matched the manipulation scenario.

#### Moderated mediation analysis

A bootstrap analysis based on 5,000 samples using the PROCESS Model 15 in SPSS (Hayes, 2013) was conducted to examine the moderated mediation effect that was whether need for uniqueness moderated the direct and indirect effects. The scarcity appeal as the independent variable (X), the perceived uniqueness as the mediator (M), purchase intention as the dependent variable (Y), and the need for uniqueness as the moderator (W).

The results showed that scarcity appeal predicted the perceived uniqueness significantly (*B* = 1.91, *SE* = 0.18, *p* < *0*.001, 95% CI [1.55, 2.27]). The interaction between perceived uniqueness and need for uniqueness on purchase intention was significant (*B* = 0.24, *SE* = 0.05, *p* < 0.001, 95% CI [0.14, 0.33]), while the interaction between scarcity appeal and need for uniqueness on purchase intention was not significant (*B* = 0.15, *SE* = 0.17, *p* = 0.037, 95% CI [−0.18, 0.48]). Thus, the need for uniqueness only moderated the indirect effect of scarcity appeal on purchase intention *via* perceived uniqueness (*B* = 0.45, *SE* = 0.09, 95% CI [0.27, 0.66]).

**Figure 2:**
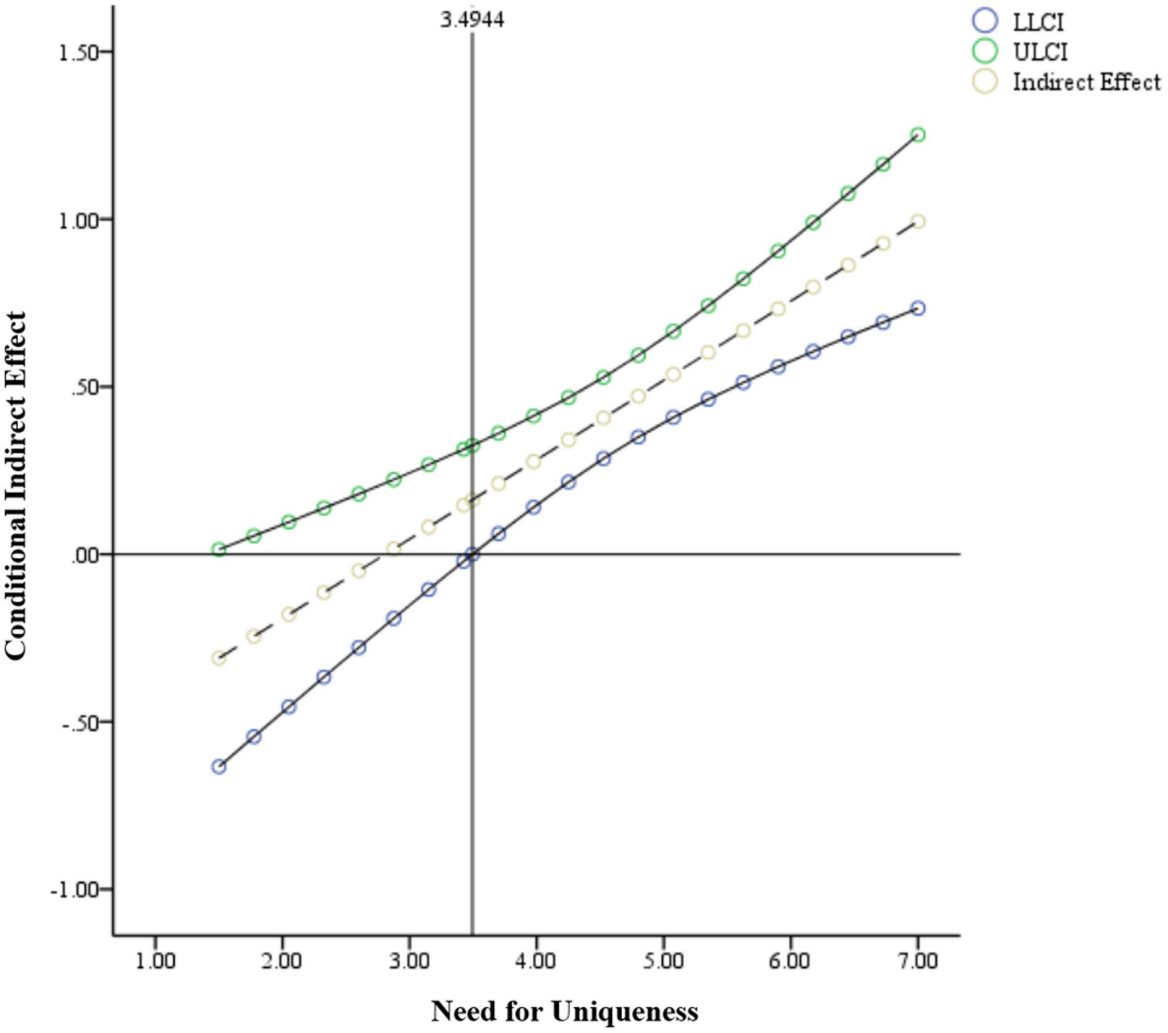
The conditional indirect effect of scarcity appeal on purchase intention *via* perceived uniqueness (moderator: need for uniqueness). The horizontal line denotes an indirect effect of zero; the vertical lines represent the boundary of the region of significance.

We used Johnson-Neyman technique (Hayes, 2018) to probe significant conditional indirect effects. We plotted the conditional indirect effect of scarcity appeal on purchase intention *via* perceived uniqueness (see Figure 2). As shown in Figure 2, significant mediation effects were observed for individuals with values of need for uniqueness were greater than 3.494 (on a 7-point scale), indicating the effect of scarcity appeal on purchase intention *via* perceived uniqueness was significant and moderated by need for uniqueness.

### Discussion

The results confirmed that the need for uniqueness moderated the effectiveness of different types of scarcity appeals. Supply-based (vs. demand-based) scarcity increased consumers’ perception of uniqueness. Perceived uniqueness increased the purchase intention of consumers with high need for uniqueness. Hence, H3 was supported.

## General discussion

Marketers often assume that scarcity appeals in terms of limited quantity positively influence purchase intentions. However, the reason behind the scarcity of the product (i.e., due to excessive demand or to restricted supply) may be as important as the scarcity itself. Existing literature provides limited guidance on using demand versus supply scarcity appeals to target different consumers. In three studies, we demonstrate that consumers perceive scarce products due to supply as more unique than scarce products due to demand. In addition, the uniqueness perception increases or decreases consumers’ purchase intention depending on their need for uniqueness. Specifically, supply-scarce products (vs. demand-scarce products) increase purchase intention for consumers with high need for uniqueness but decreases purchase intention for consumers with low need for uniqueness. The current findings have implications for researchers and practitioners interested in scarcity effects.

### Theoretical contributions

The current research offers several theoretical contributions. First, this research contributes to the literature on scarcity by highlighting the distinct impacts of supply scarcity and demand scarcity on purchase intentions. Prior research has examined the effects of product scarcity on consumer preferences (Jang et al., 2015), the origins of scarcity preferences (John et al., 2018), and the negative effects of scarcity promotions (Kristofferson et al., 2017). Researchers also examined the differential effects of limited-time scarcity (LTS) and limited-quantity scarcity (LQS) messages on conspicuous and non-conspicuous products (Jang et al., 2015). Complementing this line of research, our research makes a distinction between different sources of scarcity, namely supply induced and demand induced. This research found that consumers do differentiate between the two types of scarcity, even when the extent of scarcity is actually the same.

Second, the current research contributes to recent findings on limited-edition promotions (Tseng et al., 2021) by suggesting that supply-driven scarcity is not always effective for promoting sales. We identify need for uniqueness as a moderator of the effectiveness of supply- (vs. demand-) driven scarcity on consumers’ purchase intentions and provide process support to the mechanism. Previous works have largely focused on positive outcomes (e.g., desirability) of limited-supply scarcity appeals (e.g., Aguirre-Rodriguez, 2013). Meanwhile, recent research has identified boundary conditions under which consumers react positively to supply scarcity appeals, such as the social visibility of the product (Tseng et al., 2021) or consumers’ sense of power (Huang et al., 2020). In addition, Roy and Sharma (2015) found that consumers high in need for uniqueness trait prefer supply over demand scarcity appeal. The current research replicated the moderating role of need for uniqueness and extended the previous work by ruling out different level of scarcity as an alternative explanation. Moreover, the research suggests that supply scarcity increases purchase intentions when consumers are dispositionally and/or situationally high in need for uniqueness.

Finally, this work provides evidence for the process driving the differential effects of supply versus demand scarcity. Specifically, an important inference that consumers draw from supply- versus demand-based scarcity is the uniqueness of the product, which sequentially increases or decreases purchase intentions depending on consumers’ need for uniqueness. Previous research has shown that supply-driven scarcity messages influence consumer competition (Kristofferson et al., 2017), perceived quality (Gierl and Huettl, 2010), and risk perception (Huang et al., 2020). Another research found that supply-side scarcity appeals lead to higher perceived uniqueness than popularity (i.e., high demand) appeals (Wu and Lee, 2016). Our research further matched scarcity level and showed that scarcity type (supply versus demand) itself is enough to elicit different levels of uniqueness perception, which positively or negatively influence purchase intention. Thus, the current research adds to the scarcity appeal literature by highlighting uniqueness perception as a mechanism that encourage or discourage consumers to purchase supply scarce products.

### Practical implications

The current research provides a hint for marketers on how to select the suitable scarcity appeal. Specifically, our findings alert marketers that the effectiveness of different scarcity appeals varies as a function of consumers’ need for uniqueness. The results reveal that a supply-based scarcity appeal is more effective than a demand-based scarcity appeal for consumers with high need for uniqueness, whereas a demand-based scarcity appeal is more effective than a supply-based scarcity appeal for consumers with low need for uniqueness. Therefore, marketers need to segment and target consumers with different levels of need for uniqueness using different types of scarcity appeals. On the one hand, marketers may attempt to capture consumers’ behavior patterns and segment consumers on the basis of the predictors of need for uniqueness. On the other hand, marketers may also activate consumers’ uniqueness desire through situational primes (e.g., advertising slogans). Thus, increased need for uniqueness combined with supply-based scarcity appeals can effectively boost purchase intentions. In contrast, when consumers’ need for uniqueness cannot be identified or primed, a safe strategy is to implement demand-based scarcity appeal.

### Limitations and future research

Our research has limitations that may open avenues for future studies. First, to make the situation familiar to most consumers, we used a common product (i.e., shoes). However, the findings may not be generalizable to the wide variety of products and services. In addition, we refrained from designating specific brands for the product. This endeavor had the advantage of avoiding the confounding effects of preexisting brand knowledge and preferences. However, it also deterred us from exploring whether the current findings differed in terms of brand concepts, such as conspicuous versus non-conspicuous products, symbolic versus functional products, and hedonic versus utilitarian products (for a review, see Shi et al., 2020). Research has suggested that limited-quantity (vs. limited-time) scarcity messages affect purchase intentions more for a symbolic brand than for a functional brand (Aggarwal et al., 2011). Furthermore, we used hypothetical scenarios and measured purchase intentions, which are not equitable to actual purchasing in real situations. Future studies are needed to enhance the generalizability of our research.

Second, our work focused on examining the moderating role of need for uniqueness in the effectiveness of supply (vs. demand) scarcity appeals on purchase intentions. Future studies need to explore other potential moderators. For example, the three dimensions of need for uniqueness (i.e., creative choice, unpopular choice, and avoidance of similarity) were found to influence bandwagon and snob luxury consumption differently (Das et al., 2021). Desire for exclusivity, which emphasizes consumers’ desire to express superiority over others *via* consumption (Kim, 2018), may drive preference for limited-edition status consumption. Clues about other consumers’ behaviors (e.g., demand scarcity) appeal to consumers from a collectivist culture, whereas clues about uniqueness (e.g., supply scarcity) appeal to consumers from an individualist culture (Huang et al., 2020). Hence, these factors are likely to moderate the effects of different scarcity appeals.

Third, our research mainly focuses on perceived product uniqueness as the mechanism underlying the effects of supply (vs. demand) scarcity and need for uniqueness on purchase intentions. Prior work has found that supply scarcity appeal and demand scarcity appeal engender distinct inferences, such as differences in product quality (Gierl and Huettl, 2010) or perceived competitions among consumers (Aggarwal et al., 2011). In addition, consumers might perceive supply scarcity as artificial if they believe that a company deliberately limits the supply (Hamilton et al., 2019). Future research may examine these additional potential mechanisms, which drive the interactive effects of scarcity type and need for uniqueness.

To conclude, the current research showed the distinct impacts of supply scarcity and demand scarcity on purchase intention. The current findings provide theoretical implications on how scarcity shapes consumer responses and practical implications on how to target consumers with different levels of need for uniqueness.

## Data availability statement

The raw data supporting the conclusions of this article will be made available by the authors, without undue reservation.

## Ethics statement

The studies involving human participants were reviewed and approved by University Committee on Human Research Protection of East China Normal University. The patients/participants provided their written informed consent to participate in this study.

## Author contributions

YW, SK, and ML designed the studies and conducted analyses. SK and ML collected data. YW, SK, ML, and LL drafted the original manuscript. All authors edited and approved of the final manuscript.

## Funding

This research was financially funded by Shanghai Planning Office of Philosophy and Social Science (grant number: 2020BSH006) and the National Natural Science Foundation of China (grant number: 31600915).

## Conflict of interest

The authors declare that the research was conducted in the absence of any commercial or financial relationships that could be construed as a potential conflict of interest.

## Publisher’s note

All claims expressed in this article are solely those of the authors and do not necessarily represent those of their affiliated organizations, or those of the publisher, the editors and the reviewers. Any product that may be evaluated in this article, or claim that may be made by its manufacturer, is not guaranteed or endorsed by the publisher.

## Footnotes

^1^ https://www.wjx.cn/
